# Botanical Description of British Plants

**Published:** 1806-01

**Authors:** 


### *Botanical Description of British Plants.*

[ Continued from Vol. xiv. p. 541—546. ]

16. Polygonum. *P. persicaria.*

*Ang.* Dead, or Spotted Arse-smart. Spotted Snakeweed.

*Gen. Desc. As above.*

*Spec Desc. Stam. six; caps. one-cell. Styles* two, often cloven into three parts, and then the germen and seed three-square. *Leaves,* nearly smooth, bristled, at the last sp. *Flowers,* three or four together, in a fringed membraneous sheath, on short unequal fruit-stalks; rose-colour; segm. concave, unequal. *Ditches, waterside, cornfields.* Bl. July, Sept.

*Use.* Its taste is slighlty acid and astringent. Woollen cloth dipped in a solution of alum, obtains from this plant a yellow colour. Horses, sheep and goats eat it; cows and swine refuse it.—*Withering.*

17 Polygonum. *P. bistorta-serpentaria. Bistorta major.*

*Ang.* Great Bistort, or Snakeweed. Easter-giant, *in the North of England.*

*Gen. Desc. As above.*

*Spec. Desc. Stam. eight, pistils three, spike single, stem undivided. Leaves* egg-shaped, *root-leaves* extended along the leaf-stalk, stem-leaves sheathing the stem. *Flowers* in a spike: common cal. of two valves; within each cal. two flowers, one on a fruit-stalk, the other sitting, surrounded by a proper cup, thin, skinny, cylindrical; mouth even, entire. *Bloss.* five-div. red. *Anthers* double. *Moist meadows.* Bl. May, June.

*Use.* Every part of the plant possesses a considerable styptic power; and the root is esteemed to be one of the most powerful of the vegetable astringents. It was formerly considered to be *alexipharmic* and *sudorific,* but it is chiefly useful, on account of its styptic powers in hæmorrhages and other immoderate fluxes. It is observed by Dr. Cullen, both from its sensible qualities, from the colour it gives with green vitriol, and from the extracts it affords, to to be one of the strongest of our vegetable astringents; and to be justly commended for every virtue that had been ascribed to any other: as such, he has frequently employed it in *intermittent fevers,* both by itself, and along with gentian; he has given to the quantity of 3 drs. in a day. *Mat. Med. ii.* 40. The dose of the root in substance is from a scruple to a drachm.—*Woodville.* In the North of England, where it is known by the name of Easter-giant, the young shoots and leaves are eaten in herb pudding; and about Manchester they are substituted for greens by the name of patience dock.—*Withering.*

18. POLYGONUM. *P. aviculare.*

*Ang.* Knot grass. Snake weed.

*Gen. Desc.* *As above.*

*Spec. Desc.* *Stam. eight. Pist. three. Spike single. Stem branched. Leaves,* some egg, some spear-shaped. *Flowers* axillary, two or three together; greenish without, white within, often tinged with pink. Cal. double, skinny; outer of five segm. incloses three florets, inner only the third floret, and sometimes a rudiment of the fourth. *Fruit-stalks* short, 2 longer. *Stem.* trailing, scored, thickest at the joints. *Road sides, paths, streets, corn fields.* BL. April, Sept.

*Use.* The seeds are useful for every purpose in which those of the next species are employed. Great numbers of small birds, especially sparrows, feed on them.—*Withering.*—The stubbles in Sweden are purpled over with this plant.—*Linn.*

19. POLYGONUM. *P. fagopyrum. Helsinecaule erecto, Erysinum vulgare.*

*Ang.* Buck-wheat. Snake-weed. Bucke. Branks. French wheat. Crap.

*Gen Desc.* *As Above.*

*Spec. Desc.* *Leaves* heart-arrow-shaped. *Stem* nearly upright, without prickles, angles of the seeds equal. *Bloss.* purplish white. *Spikes* shorter than the leaves. *In Corn fields.* BL. July August.

*Use.* The seeds furnish a nutritious meal, which is not apt to turn acid upon the stomach; it is made into cakes, in some parts of England, called *crumpits.* These seeds are an excellent food for poultry. The flowers afford good food for bees, and as they appear late in the summer, M. du Hamel, in his Observations on the management of Bees, advises the removal of the hives in autumn to a situation in the vicinity of this plant. Sheep that eat it become unhealthy. It is usual with farmers to sow a crop of buck-wheat, and plow it under when fully grown, as a manure for the land. It is very impatient of cold, dying at the very first attack of frost. Cows, sheep, and goats eat it; horses and swine refuse it.—*Withering.*

20. POLYGONUM. *P. convolvulus. Tragopyrum scandens- Convolvulus minor.*

*Ang.* Black bind-weed. Climbing snake-weed.

*Gen. Desc. As above.*

*Spec. Desc. Leaves* heart or arrow-shaped; stem twining, angular. *Spikes* longer than the leaves. *Flowers* blunted, greenish. *Anthers* red. *Corn fields, hedges.* BL. June, Sept.

*Use.* The seeds are perfectly as good for use as those of the preceding species, but they are produced in greater quantity, and this plant bears cold better.—*Withering.* Cows and goats eat it; sheep, swine, and horses refuse it. *Linn.* A horse eat it.—*Sl.*

OCTANDRIA. TETRAGYNIA.

21. PARIS. *P. quadrifolia. Solanum quadrifolium facciferum.*

*Ang.* Herb Paris. True love. One-berry. Four-leaved true love.

*Gen. Desc.* Calyx four-leaved. Petals four, narrower. Berry four-celled.

*Spec. Desc. Stem-leaves* generally four, at the top of the stalk, eggs-haped, tapering, shining, from one to seven. *Stem* naked. *Root* fleshy. *Bloss.* pale green. *Cal.* with only three leaves. *Styles* purplish black. *Woods, shady places.* BL. May, June.

*Use.* The leaves and berries are said to partake of the properties of opium. The juice of the berries is useful in inflammations of the eyes. Linnæus says, that the roots will vomit as well as ipecacuanha, but must be given in double quantity.—*Withering.*

22. QUERCUS. *Q. robur. Q. vulgare. Q. latifolia.*

*Ang.* Oak.

*Gen. Desc.* M. and Fem. fl. on the same plant. Bloss. O. M. cal. five cleft. Stem five to ten. F. cal. one leaf, very entire, rough. Styles two to five, nut egg-shaped, leather-like, of one seed, and, when ripe, of one cell.

*Spec. Desc. Leaves* on leaf-stalks, oblong, broadest toward the end, indentations rather acute, angles, blunt, deep green; frequently remain all winter. *Fruit* nearly sitting, *acorns* four to twelve together, egg-shaped, sometimes oblong, taper pointed. *Wood,* reddish, brittle. *Common*

*mon*

*mon, but less so than the* Quercus foemina, *of which the following is the*

*Spec. Desc. Leaves* oblong, on short leaf-stalks, blunt, wing cleft, with indentations, but not so deep or so irregular as the preceding, opposite deeper green, narrower towards the end. *Fruit* mostly solitary, on long fruit-stalks, or from one to three together. M. fl. *stam.* mostly 8; cal. six to eight-cleft, according to the number of stamens. Fem. fl. cal. tiled, about twenty leaves; *styles* mostly three, bent back, *wood* whitish, hard. The leaves, flower and fruit, later than the preceding. *Woods and hedges.* Bl. April.

*Use.* The following remarks apply equally to both these species. The bark is the part directed for medicinal use; (by the ancients various parts of the tree were used;) it manifests to the taste a strong astringency, accompanied with a moderate bitterness: its universal use and preference in tanning of leather is a proof of its great astringency, and, like other astringents, it has been recommended in *Agues*, and for restraining *hœmorrhagies*, *alvine fluxes*, and other immoderate evacuations. A decoction of it has likewise been advantageously employed as a gargle, and as a fomentation or lotion, in *procidentia recti et uteri.* Dr. Cullen has frequently used the decoction with success in slight tumefactions of the mucous membrane of the fauces, and in *prolapsus uvulœ* and *cynanche tonsillaris*, to which some are liable on the least exposure to cold: in many cases, this decoction, early applied, has served to prevent these disorders. Dr. C. however, constantly added a portion of alum, but he tried also a solution of alum alone, and "it did not prove so effectual." M. M. ii. 45. This bark has been supposed not less efficacious than that of cinchona; but this opinion now obtains little credit, though there be no doubt that oak bark may have the power of curing *intermittents.* Dr. Cullen "gave it in powder, half a drachm every two or three hours during the intermissions of a fever, and both by itself, and joined with camomile flowers, prevented the return of the paroxysms of intermittents.—*Cullen.* Galls, which are found upon the leaves of this tree, are the most powerful of the vegetable astringents, striking a deep black when mixed with a solution of ferrum vitriolatum, and therefore preferred to every other substance for making ink. As a medicine, they are to be considered as applicable to the same indications as the bark of this tree; and by possessing a greater degree of astringent and styptic

 power, power, seem to be better suited to external use. Reduced to a fine powder and made into an ointment, they have been found of great service in *hæmorrhoidal* affections.—*Cullen.* Their efficacy in *intermittents* has also been tried, and in many cases they succeeded, but in many others they failed, which were afterwards cured by the Peruvian bark. *See Mem. de l'Acad.* des Sc. 1702.—*Woodville.* Oak saw-dust is the principal indigenous vegetable used in dying fustian: all the varieties of drabs and different shades of brown, are made with oak saw-dust, variously managed and compounded. The balls or oak-apples are likewise used in dying, as a substitute for galls. The *black* obtained from them by the addition of copperas, is more beautiful but less durable than that from galls. The bark is universally used to tan leather: an infusion of it, with a small quantity of copperas, is used by the common people to dye woollens of a *purplish blue;* the colour, though not bright, is durable. The wood is hard, tough, tolerably flexible, and does not easily splinter; it is therefore preferred to all other timber for ship building: it is well adapted to almost every purpose of the carpenter; but to enumerate all its uses would be superfluous and difficult. Horses, cows, sheep, and goats eat the leaves; swine and deer fatten on the acorns.—*Withering.*

23. RHODIOLA. *R. rosea.*

*Ang.* Yellow rose-wort. Rose-root.

*Gen. Desc.* M. and Fem. fl. on different plants. Cal. four-div. M. bloss. four-petal. Fem. bloss. 0. Nectaries four. Caps. four, many-seeded.

*Spec. Desc. Stam.* six, eight, or twelve, longer than the blossom. *Stems* numerous, simple, four to ten inches high, cylindrical, smooth, hollow, leafy. *Leaves* numerous, egg and egg-spear-shaped, serrated above, at the base entire, fleshy, sea-green, sometimes tinged with purple. *Root* white. *Styles* short, pointing outwards, permanent. *Bloss.* terminating yellow. *Mountains, rocks.* BL. June, July.

*Use.* The root has the fragrance of a rose, particularly when dried, but if cultivated in a garden it loses most of its sweetness.—*Withering.* The inhabitants of the Farro Islands use this plant as a remedy for the *scurvy.* A cataplasm of the fresh roots applied to the forehead, is said to relieve the head-ach, and to heal malignant ulcers. In Greenland this plant is eaten as garden stuff. A fragrant rose water is distilled from the root.—*Lightfoot.* Sheep and goats eat it; cows and swine refuse it.

### CLASS IX. ENNEANDRIA.

### Enneandria. Digynia.

1. MERCURIALIS. *M. perennis.*

*Ang.* Dog's Mercury.

*Gen Desc.* M. and F. fl. on different plants; bloss. 0. cal. 3 div. M. stamens nine to twelve. Anthers globular, double. F. caps. double, two-celled, one seed in each.

*Spec Desc.* *Stem* undivided; *leaves* rough. *Male spikes* longer than the leaves, *Fem. sp.* shorter. *M. flowers* in spikes, axillery. *Fem. fl.* on fruit-stalks, generally solitary. *Anthers,* two on each filam. *Flowers* yellow green. *Woods, hedges-banks.* BL. Ap. May.

*Use.* This plant is of a soporific deleterious nature, noxious both to man and beast: it is sometimes eaten in mistake for chenopodium bonus-henricus, with fatal effects. In the Isle of Skye, it is said to be used sometimes, taken in infusion, in order to bring on *salivation:* "how it answers I know not, but the experiment seems dangerous." *Lightfoot.* This plant is noxious to sheep, and deleterious to man. Ray relates the case of a man, his wife, and three children, who experienced deleterious effects from eating it fried with bacon. In drying it turns blue, and steeped in water, affords a fine deep blue colour, but which unfortunately is destroyed by acids and alkalies, and not recoverable by any means yet found out. Sheep and goats eat it, horses and cows refuse it.—*Withering.*

2. MERCURIALIS. *M. annua.*

*Ang.* French Mercury. Annual Mercury.

*Gen. Desc.* *As above.*

*Spec. Desc.* *Stem* branching; *leaves* smooth; *flowers* in spikes; barren sp. shorter than the fertile; M. calx. smooth; F. cal. hairy. *Stam.* sometimes 16. *Waste-places and dunghills, about towns and villages.* BL. Aug. Sept.

*Use.* The whole plant is mucilaginous, and was formerly much employed as an emollient, but is now disregarded. The seeds taste like those of hemp.—*Withering.* Tournefort says that the French make a syrup of the juice, of which 2 oz. is given for a purge, and that they use it in clysters and pessaries, mixing two thirds of honey, or 1 part to 1 ½ of the juice. In England it is out of use.—*Lightfoot.*

### CLASS X. DECANDRIA.

### Decandria. Monogynia.

1. ARBUTUS. *A. unedo.*

*Ang.* Arbutus, Strawberry-tree.

*Gen. Desc.* Cal. five-div. bloss; egg-shaped, pellucid at the base; mouth five-cleft. Berry five celled, many seeded, superior.

*Spec. Desc. Stem* tree-like. *Leaves,* smooth, bluntly serrated, serratures coloured. *Panicle* terminating. Cal. segm. lapping over each other, coloured at the points. *Bloss.* White, a little hairy within. *Anthers* scarlet, double, opening at the base, with two yellow horns. Berries red, rough with tubercles formed by the seeds. *West of Ireland.* Bl. Sept.

*Use.* The fruit, though pleasing to the eye, is not grateful to the taste; the country people however in Ireland are said to eat it, but they always drink water after; it grows chiefly on barren rocks, about the Lake of Killarney, in the county of Kerry, where it arrives at a very large size. It is a beautiful ornament to our shrubberies on account both of the foliage, the flowers, and the fruit; the latter resembles a strawberry.

2. Arbutus. *A. alpania.*

*Ang.* Mountain Strawberry-tree.

*Gen. Desc. As above.*

*Spec. Desc. Stem* trailing. *Leaves* wrinkled, somewhat serrated, fringed. *Berries* black, globular, sitting a very small red cup. *Dry Heaths in Scotland.* Bl. May.

*Use.* The berries of this species of Arbutus, have something of the flavor of black currants, but they are not so good.— Goats refuse it.—*Withering.*

( To be continued. )

